# 7-Iodo-5-aza-7-deazaguanine ribonucleoside: crystal structure, physical properties, base-pair stability and functionalization

**DOI:** 10.1107/S2053229620004684

**Published:** 2020-04-29

**Authors:** Dasharath Kondhare, Simone Budow-Busse, Constantin Daniliuc, Frank Seela

**Affiliations:** a Laboratory of Bioorganic Chemistry and Chemical Biology, Center for Nanotechnology, Heisenbergstrasse 11, 48149 Münster, Germany; b Organisch-Chemisches Institut, Westfälische Wilhelms-Universität Münster, Corrensstrasse 40, 48149 Münster, Germany; c Laboratorium für Organische und Bioorganische Chemie, Institut für Chemie, Universität Osnabrück, Barbarastrasse 7, 49069 Osnabrück, Germany

**Keywords:** 7-iodo-5-aza-7-de­aza­guanosine, ribonucleoside, crystal structure, Hirshfeld surface analysis, base-pair prediction, crystal packing, all-purine RNA, p*K_a_* values

## Abstract

The positional change of nitro­gen-7 of the RNA constituent guanosine to the bridgehead position-5 leads to the base-modified nucleoside 5-aza-7-de­aza­guanosine. Contrary to guanosine, this mol­ecule cannot form Hoogsteen base pairs and the Watson–Crick proton donor site N3—H becomes a proton-acceptor site. This causes changes in nucleobase recognition in nucleic acids and has been used to construct stable ‘all-purine’ DNA and DNA with silver-mediated base pairs. The present work reports the single-crystal X-ray structure of 7-iodo-5-aza-7-de­aza­guanosine, C_10_H_12_IN_5_O_5_ (**1**). The iodinated nucleoside shows an *anti* conformation at the glycosylic bond and an *N* conformation (O4′-*endo*) for the ribose moiety, with an anti­periplanar orientation of the 5′-hy­droxy group. Crystal packing is controlled by inter­actions between nucleobase and sugar moieties. The 7-iodo substituent forms a contact to oxygen-2′ of the ribose moiety. Self-pairing of the nucleobases does not take place. A Hirshfeld surface analysis of **1** highlights the contacts of the nucleobase and sugar moiety (O—H⋯O and N—H⋯O). The concept of p*K*-value differences to evaluate base-pair stability was applied to purine–purine base pairing and stable base pairs were predicted for the construction of ‘all-purine’ RNA. Furthermore, the 7-iodo substituent of **1** was functionalized with benzo­furan to detect motional constraints by fluorescence spectroscopy.

## Abstract 2

7-Iodo-5-aza-7-de­aza­guanosine represents a base-modified nucleoside with an entirely different recognition pattern as the parent purine com­pound. It shows an *anti* conformation at the glycosylic bond and an *N* conformation (O4′-*endo*) for the ribose moiety. Packing is controlled by hydrogen bonds and by a 7-iodo contact to 2′-oxygen of the ribose moiety.

## Introduction   

**Figure 1:**
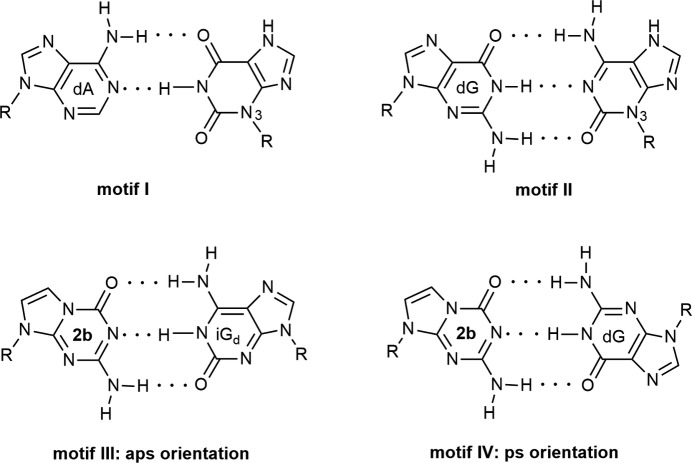
Upper row: ‘all-purine’ pairs suggested by Wächtershäuser (1988 ▸). Lower row: purine–purine base pairing motifs of 5-aza-7-de­aza­guanine 2′-de­oxyribonucleoside (**2b**) with anti­parallel (aps) or parallel (ps) strand orientation (Seela *et al.*, 2001 ▸). Notes: iG_d_ = 2′-de­oxy­isoguanosine, dG = 2′-de­oxy­guanosine and R = β-d-2′-de­oxy­ribo­furanosyl.

The specific recognition of com­plementary nucleobases in the DNA coding system is based on the combination of purine and pyrimidine bases (Watson & Crick, 1953 ▸). Adenine pairs with thymine and guanine with cytosine. Nevertheless, an alternative coding system entirely com­posed of purines (‘all-purine’ DNA) was suggested by Wächtershäuser (1988 ▸). In this, purines glycosyl­ated at nitro­gen-9 (adenine and guanine) base pair with purines glycosyl­ated at nitro­gen-3 (motif I xanthine and motif II isoguanine, see Fig. 1 ▸; purine numbering is used throughout this article). The resulting purine–purine base pairs are isoelectronic and isogeometric to standard purine–pyrimidine Watson–Crick pairs. However, purines glycosyl­ated at nitro­gen-3 are unstable and difficult to handle (Leonard, 2020 ▸).

**Figure 2:**
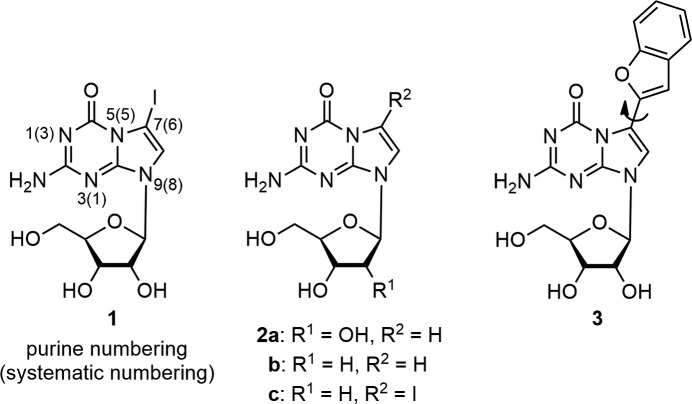
5-Aza-7-de­aza­guanine nucleosides.

Recent advances in the construction of ‘all-purine’ DNA used purine nucleosides glycosyl­ated at nitro­gen-9 (Seela *et al.*, 1997 ▸, 2001 ▸; Seela & Melenewski, 1999 ▸; Seela & Rosemeyer, 2002 ▸). In this context, 5-aza-7-de­aza­guanine (imidazo[1,2-*a*]-1,3,5-triazine) 2′-de­oxy­ribonucleoside (**2b**) has emer­ged as a promising purine analogue mimicking the pyrimidine site of a Watson–Crick base pair (Fig. 2 ▸). More specifically, 5-aza-7-de­aza­guanine is able to pair with isoguanine or gua­nine (Seela & Rosemeyer, 2002 ▸). The 5-aza-7-de­aza­guanine–isoguanine base pair forms stable ‘all-purine’ DNA with an anti­parallel strand orientation (motif III, Fig. 1 ▸), whereas the 5-aza-7-de­aza­guanine–guanine pair leads to DNA with parallel strands (motif IV, Fig. 1 ▸). Furthermore, a con­figur­ational change at the anomeric centre from β-d to α-d resulted in heterochiral ‘all-purine’ DNA causing an additional change of the strand orientation (Seela *et al.*, 2001 ▸). Size expansion of the double helix was studied with tricyclic purine bases (Winnacker & Kool, 2013 ▸).
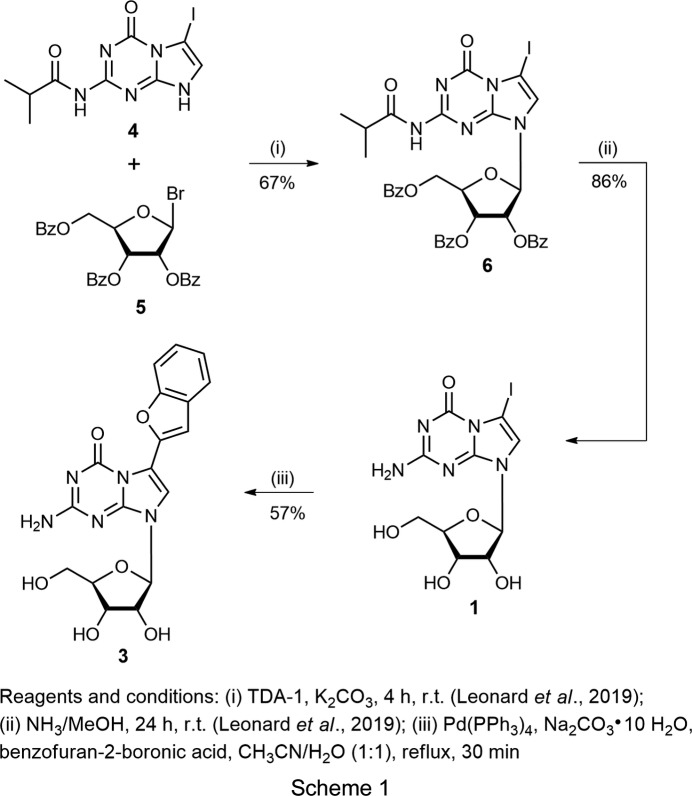


Contrary to the work performed on ‘all-purine’ DNA, very little is known in the realm of ‘all-purine’ RNA. Iodinated 5-aza-7-de­aza­guanine ribonucleoside **1** represents a convertible key com­pound within a series of 5-aza-7-de­aza­guanine nucleosides such as **2a**–**2c** (Fig. 2 ▸). It has the capability to be functionalized at position-7 of the nucleobase with various functional groups and can be cross-linked at this position. Nucleosides **1** and **2a** represent building blocks for the construction of ‘all-purine’ RNA. This work reports the single-crystal X-ray structure of the 7-iodo ribonucleoside **1** (Fig. 2 ▸) and its physical properties. The crystal packing has been studied and a Hirshfeld surface analysis performed to visualize packing inter­actions. Base-pair stabilities are predicted on the basis of differences of the p*K_a_* values (Krishnamurthy, 2012 ▸). Functionalization of **1** with a fluorescent group by cross-coupling has been performed and fluorescence data are collected.

## Experimental   

### Synthesis and crystallization of nucleoside 1   

7-Iodo-5-aza-7-de­aza­guanine (**1**) was synthesized as re­ported previously (Leonard *et al.*, 2019 ▸). For crystallization, com­pound **1** was dissolved in a methanol/water (1:1 *v*/*v*) mixture (10 mg in 1 ml) and was obtained as colourless thin needles (m.p. 180–181 °C, decom­position) by slow evaporation of the solvent (room temperature, 24 h). A colourless needle-like specimen of **1** was used for the X-ray crystallographic analysis.

### X-ray diffraction and refinement   

Crystal data, data collection and structure refinement details are summarized in Table 1 ▸. The H atoms on N2, O2′, O3′ and O5′ were refined freely, but with N—H and O—H distance restraints and *U*_iso_(H) values fixed. For atom C1′, the anisotropic displacement parameters were refined with damp­ing in order to hold this atom positive definite.

### Synthesis of benzo­furan conjugate 3   

For the preparation of 2-amino-6-(benzo­furan-2-yl)-8-(*β*-d-ribo­furanos­yl)-8*H*-imidazo[1,2-*a*]-*s*-triazin-4-one (**3**), com­pound **1** (Leonard *et al.*, 2019 ▸; 100 mg, 0.24 mmol), Pd(PPh_3_)_4_ (28 mg, 0.024 mmol), Na_2_CO_3_·10H_2_O (352 mg, 1.23 mmol) and benzo­furan-2-boronic acid (158 mg, 0.97 mmol) in CH_3_CN/H_2_O (1:1 *v*/*v*, 20 ml) were refluxed for 30 min. After consumption of starting material **1** [monitored by thin-layer chromatography (TLC)], the solvent of the reaction mixture was evaporated under reduced pressure, and the residue was applied to flash chromatography (CH_2_Cl_2_/MeOH, 90:10→75:25 *v*/*v*). Com­pound **3** was obtained from the main zone as a white solid (yield 55 mg, 57%). TLC (silica gel, CH_2_Cl_2_/MeOH, 85:15 *v*/*v*) *R*_F_ 0.4. *λ*_max_ (MeOH)/nm 312 (∊/dm^3^ mol^−1^ cm^−1^, 22600), 327 (18800). ^1^H NMR (600 MHz, DMSO-*d*_6_): *δ* 8.00 (*s*, 1H, H-8), 7.71–7.65 (*m*, 2H, benzofuran Ar-H), 7.54 (*dq*, *J* = 8.4, 1.0 Hz, 1H, benzofuran Ar-H), 7.33 (*ddd*, *J* = 8.4, 7.2, 1.3 Hz, 1H, benzofuran Ar-H), 7.26 (*td*, *J* = 7.4, 1.0 Hz, 1H, benzofuran Ar-H), 7.12 (*s*, 2H, NH_2_), 5.87 (*d*, *J* = 5.8 Hz, 1H, H-1′), 5.51 (*d*, *J* = 5.5 Hz, 1H, OH-2′), 5.20 (*d*, *J* = 4.6 Hz, 1H, OH-3′), 5.16 (*t*, *J* = 5.3 Hz, 1H, OH-5′), 4.37 (*q*, *J* = 5.4 Hz, 1H, H-2′), 4.11 (*td*, *J* = 4.7, 3.4 Hz, 1H, H-3′), 3.92 (*q*, *J* = 3.6 Hz, 1H, H-4′), 3.66 (*ddd*, *J* = 11.9, 5.2, 3.8 Hz, 1H, H-5′), 3.58 (*ddd*, *J* = 11.9, 5.3, 3.6 Hz, 1H, H-5′). ^13^C NMR (151 MHz, DMSO-*d*_6_): *δ* 164.8, 153.9, 151.9, 150.1, 144.7, 128.4, 125.1, 123.2, 121.6, 115.7, 114.7, 110.7, 108.2, 86.5, 85.5, 85.4, 73.7, 70.2, 61.1, 61.0. HRMS (ESI–TOF) *m*/*z*: [*M* + Na^+^] calculated for C_18_H_17_N_5_NaO_6_, 422.1071; found, 422.1075.

## Results and discussion   

### Mol­ecular geometry and conformation of 7-iodo-5-aza-7-de­aza­guanosine (1)   

**Figure 3:**
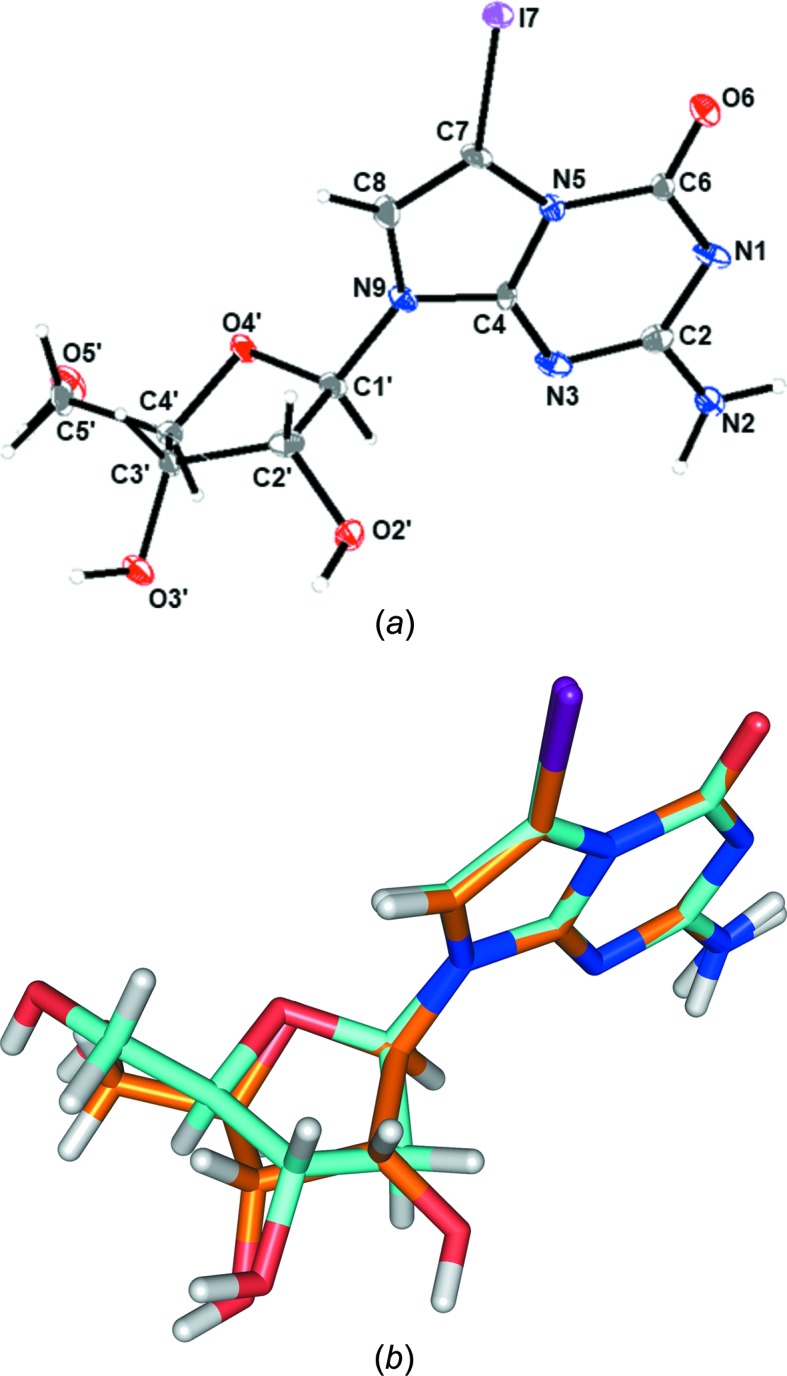
(*a*) Perspective view of **1**, showing the atom-numbering scheme. Displacement ellipsoids are drawn at the 50% probability level and H atoms are shown as small spheres of arbitrary size. (*b*) Overlay of ribonucleoside **1** and the related 2′-de­oxy­ribonucleoside **2c**.

The three-dimensional (3D) structure of **1** is shown in Fig. 3 ▸(*a*) and selected crystallographic data and structure refinement details are summarized in Table 1 ▸. According to the Flack parameter and the synthetic pathway, the anomeric centre at C1′ is in an *R* configuration confirming the β-d anomeric structure of **1**. The structure of the related 2′-de­oxy­ribonucleoside has been reported previously (for selected geometric parameters of the single-crystal X-ray analysis of **2c**, see Table S1 in the supporting information) (Leonard *et al.*, 2019 ▸).

To evaluate the impact of the 2′-hy­droxy group of ribonucleoside **1** on the crystal structure, it was of inter­est to com­pare the geometric parameters of com­pound **1** with those of the 2′-de­oxy­ribonucleoside **2c** (Leonard *et al.*, 2019 ▸). An overlay of both mol­ecules visualizes the conformational differences mainly affecting the sugar moiety (Fig. 3 ▸ *b*).

The nucleobase may adopt two principal orientations with respect to the sugar moiety (*syn*/*anti*) and it is defined by the torsion angle χ (O4′—C1′—N9—C4) (IUPAC–IUB Joint Commission on Biochemical Nomenclature, 1983 ▸). Purine nucleosides are found in a wide range of *anti* conformations (Blackburn *et al.*, 2006 ▸). The 7-iodo-5-aza-7-de­aza­guanine moiety of both nucleosides also adopts an *anti* conformation, with χ = −120.6 (9)° for ribonucleoside **1** (Table 2 ▸) and χ = −139.9 (6)° for **2c** (Leonard *et al.*, 2019 ▸). The glycosylic bond lengths of **1** (Table 2 ▸) and **2c** (Lin *et al.*, 2004 ▸) are within the range of purine nucleosides (Saenger, 1984 ▸).

The ribose ring is twisted out of plane to minimize steric inter­actions of the substituents, referred to as sugar puckering (Altona & Sundaralingam, 1972 ▸). The more electronegative substituents of C2′ and C3′ prefer an axial orientation. Nucleosides can adopt two principal sugar puckering modes, namely C3′-*endo* (*N*) and C2′-*endo* (*S*) corresponding to the major displacement of C3′ or C2′ from the median C1′/O4′/C4′ plane. The C2′-*endo* conformation is the preferred puckering mode of 2′-de­oxy­ribonucleosides (Saenger, 1984 ▸), whereas ribonucleosides show a preference for the C3′-*endo* sugar pucker. Also, 7-iodo ribonucleoside **1** shows an *N* conformation, with a pseudorotational phase angle *P* = 77.87° and a maximum amplitude τ_m_ = 47.14°. However, the typical C3′-*endo* conformation is less pronounced and the sugar pucker is shifted towards O4′-*endo*. This is a sugar conformation where the nucleobase and the exocyclic 5′-OH group are arranged equatorially. The sugar moiety of 2′-de­oxy­ribonucleoside **2c** also adopts an *N*-type sugar conformation (C3′-*endo*–C4′-*exo*, ^3^ *T*_4_, *P* = 28.55° and τ_m_ = 34.28°) (Leonard *et al.*, 2019 ▸).

In many cases, the solid-state conformation of nucleosides differs from that in solution and is also different from that of nucleosides as constituents of a single- or double-stranded nucleic acid. Sugar puckering of **1** was determined in solution from the coupling constants of high-resolution ^1^H NMR spectra (600 MHz) (for details, see Table S2 in the supporting information). The population of *S versus N* conformers was calculated on the basis of coupling constants using the program *PSEUROT* (Version 6.3; Van Wijk *et al.*, 1999 ▸). Notably, the conformation found for ribonucleoside **1** in solution is *S*-type (69%) and is different from that in the solid state (*N*-type). The closely related 2′-de­oxy­ribonucleoside **2c** also prefers an *S* conformation (62%) in solution (Lin *et al.*, 2004 ▸).

The orientation of the exocyclic 5′-hy­droxy group relative to the sugar ring is described by the torsion angle γ (O5′—C5′—C4′—C3′). Surprisingly, γ is identical in both nucleosides despite the fact of their having different sugar moieties (ribose *versus* 2′-de­oxy­ribose). The 5′-hy­droxy group adopts, in both cases, an anti­periplanar (*trans*) conformation, with γ = −172.9 (8)° for **1** and γ = −172.7 (4)° for **2c** (Leonard *et al.*, 2019 ▸).

### Hydrogen bonding and mol­ecular packing   

**Figure 4:**
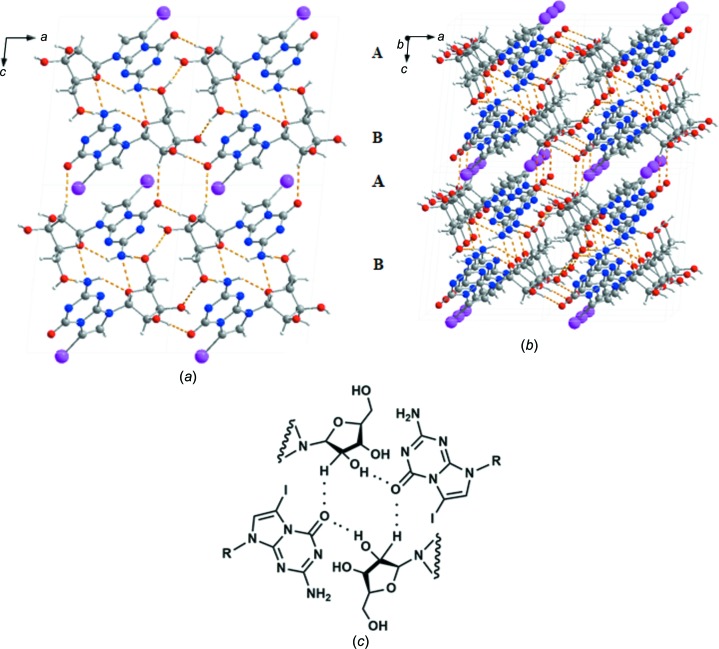
(*a*) Hydrogen bonding of **1**, viewed along the *b* axis. (*b*) Multilayered mol­ecular packing of **1**, shown along the slightly inclined *b* axis. (*c*) Detailed view of part (*a*), showing the nucleobase–sugar inter­action forming a central core.

In nucleoside solid-state structures, hydrogen bonds are formed between N and/or O atoms functioning as proton donors or acceptors. They can be provided by the nucleobases and/or the sugar moieties. Fig. 4 ▸(*a*) displays the hydrogen-bonding pattern of 7-iodo-5-aza-7-de­aza­guanosine (**1**) within one particular layer.

Nucleobase-to-nucleobase inter­actions, such as self-pairing in a Watson–Crick-like fashion, are not observed for ribonucleoside **1**. This is different from the solid-state structure of the 5-aza-7-de­aza­guanine nucleobase reported recently (Laos *et al.*, 2019 ▸). The free nucleobase is able to adopt tautomeric forms with the H atom located either at N9 or at N3. The N9—H tautomer can base pair with the N3—H tautomer, forming a tridentate base pair (Laos *et al.*, 2019 ▸). However, in the context of DNA or RNA structure, this is an artificial situation, as nucleosides glycosyl­ated at N9 lose the proton at the heterocycle and thus the ability to form these tautomers.

Com­pound **1** forms in the crystal a core of four mol­ecules displaying a ‘parallelogram’-like arrangement, with the O2′—H2′ and C2′—H2′1 groups as proton donors and atom O6 of the nucleobase as a bi-acceptor (Fig. 4 ▸ *a* and Table 3 ▸). This structure is surrounded by ‘cyclo­hexa­ne’-like motifs formed by two mol­ecules connected *via* N2—H2*A*⋯O4′^i^ and C1′—H1′⋯N2^v^ hydrogen bonds. Further stabilization of the solid-state structure is achieved by hydrogen bonds formed by the exocyclic 5′-OH group (N2—H2*B*⋯O5′^ii^ and O5′—H5′⋯O3′^iv^).

It is well documented that the iodo substituents of halogenated nucleosides can participate in mol­ecular inter­actions, sometimes even playing a predominant role. Strong iodo–iodo inter­actions have been reported for 5-iodo-α-2′-deoxycytidine (Müller *et al.*, 2019 ▸), while the iodo substituent of 2′-de­oxy­ribonucleoside **2c** shows a strong contact to atom O3′ [I7⋯O3′ = 2.794 (4) Å and C7—I7⋯O3′ = 169.2 (2)°] (Leonard *et al.*, 2019 ▸). In the crystal structure of 7-iodo-5-aza-7-de­aza­guanosine (**1**), the iodo substituent I7 shows a contact to atom O2′ [2.936 (7) Å and C7—I7⋯O2′^vi^ = 170.4 (3)°; for symmetry code, see Table 3 ▸]. However, the I⋯O contact of ribonucleoside **1** is less strong than that in com­pound **2c**.

**Figure 5:**
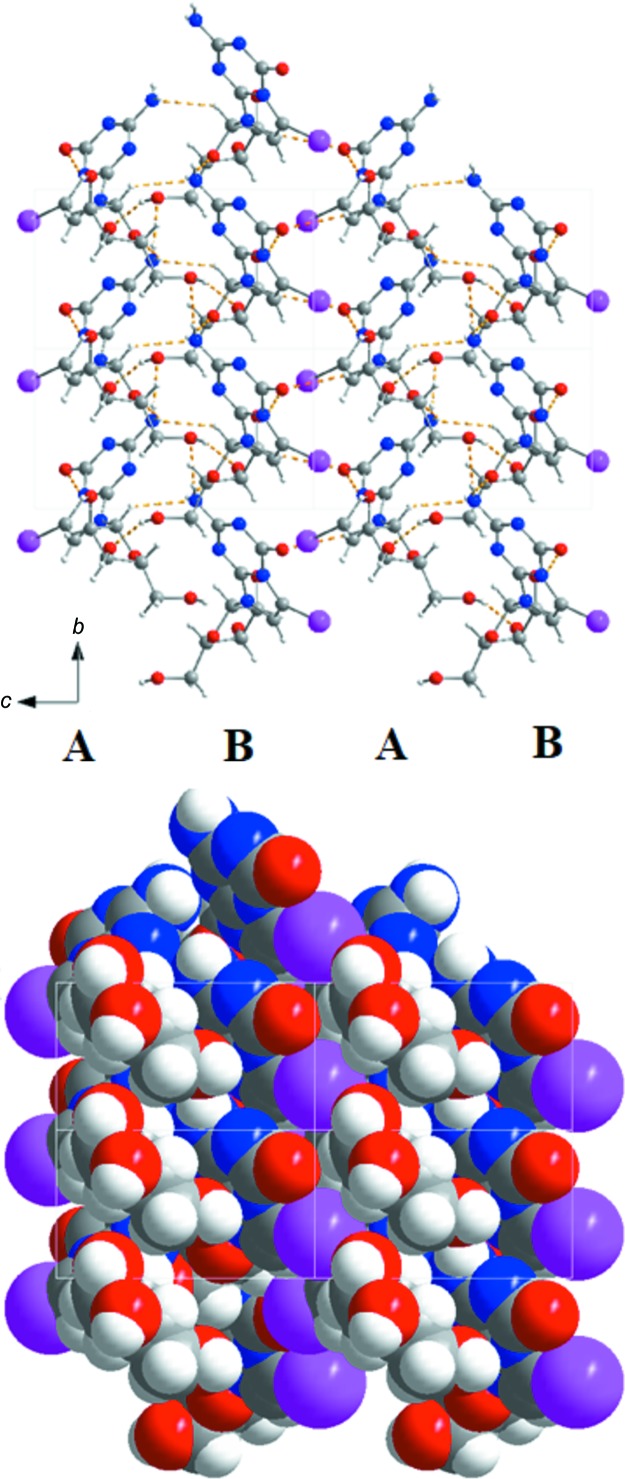
(Top) The crystal packing of **1**, showing the hydrogen bonds along the *a* axis. (Bottom) Space-filling model of **1**, viewed along the *a* axis.

The individual nucleoside layers are stacked upon each other in a very regular fashion, as demonstrated in Fig. 4 ▸(*b*). Fig. 5 ▸ shows that com­pound **1** forms chains which are arranged in an alternating reverse fashion (*A* *B* *A* *B*…). Two chains (*A* and *B*) are connected to each other by hydrogen bonds (C1′—H1′⋯N2^v^, N2—H2*B*⋯O5′^ii^ and O5′—H5′⋯O3′^iv^; Table 3 ▸). However, as was discussed above, the ribonucleoside mol­ecules are not arranged in a Watson–Crick-like fashion. Instead, the nucleobase of one chain is placed opposite the sugar residue of the next chain. As a result, the iodo substituent points towoards the outside of each double chain (com­posed of *A* and *B* chains). The double chains are connected to each other by the C2′—H2′1⋯O6^vi^ hydrogen bond. This arrangement can also be visualized by the space-filling model shown in Fig. 5 ▸.

### Hirshfeld surface analysis of 1   

**Figure 6:**
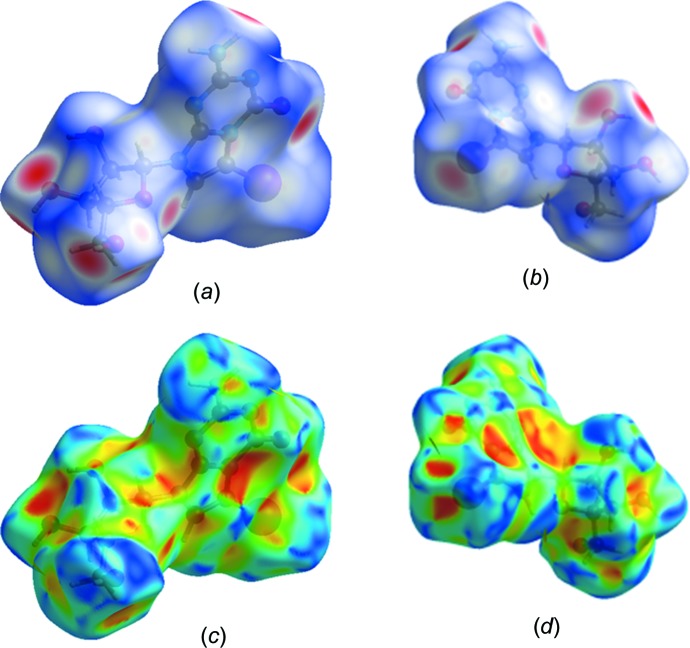
Hirshfeld surface of com­pound **1**, mapped over *d*_norm_ (−0.5 to 1.5 Å), shown in (*a*) a front and (*b*) a back view. The shape index, shown in (*c*) a front and (*d*) a back view.

Hirshfeld surface analysis is a valuable tool to obtain additional insight into the role of crystal packing forces and to visualize the inter­molecular inter­actions of crystalline com­pounds. The *CrystalExplorer* program (Version 17; Spackman & Jayatilaka, 2009 ▸; Turner *et al.*, 2017 ▸) was used to conduct a Hirshfeld surface analysis mapped over *d*_norm_ (−0.5 to 1.5 Å), shape index (−1.0 to 1.0 Å) and curvedness (−4.0 to 0.4 Å) (Fig. S2 in the supporting information), as well as a two-dimensional (2D) fingerprint plot analysis. The Hirshfeld surface of com­pound **1** is shown in Figs. 6 ▸(*a*) and 6(*b*). Several red spots are visible on the Hirshfeld surface corresponding to the close O—H⋯O and N—H⋯O contacts of the nucleobase and sugar moieties. The shape index indicates π–π stacking inter­actions by the presence of adjacent red and blue triangles. Their absence clearly suggests that there are no π–π inter­actions in com­pound **1**, as demonstrated by Figs. 6 ▸(*c*) and 6(*d*).

**Figure 7:**
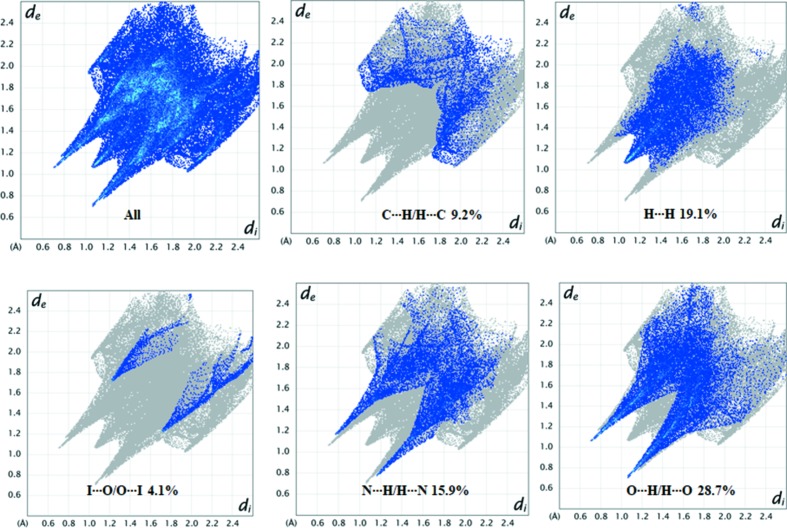
2D fingerprint plots showing the percentages of the contribution of various interactions to the total Hirshfeld surface area of com­pound **1**. Full inter­actions (upper row, left) and resolved contacts (upper row: middle, C⋯H/H⋯C; right, H⋯H; lower row: left, I⋯O/O⋯I; middle, N⋯H/H⋯N; right, O⋯H/H⋯O).

2D fingerprint plots can be resolved to highlight particular atom pair inter­actions. This enables the separation of contributions from different contact types that overlap in the full fingerprint plot. The overall 2D fingerprint plot and those resolved into H⋯C/C⋯H, H⋯H, I⋯O/O⋯I, N⋯H/H⋯N and O⋯H/H⋯O contacts are illustrated in Fig. 7 ▸, together with their relative contributions to the Hirshfeld surface. The proportions of O⋯H/H⋯O, H⋯H and N⋯H/H⋯N inter­actions com­prise 28.7, 19.1 and 15.9%, respectively, of the total Hirshfeld surfaces. Lower percentages are found for the H⋯C/C⋯H contacts (9.2%). Moreover, the inter­action between iodo substituent I7 and atom O2′ is confirmed and appears as two wings in the fingerprint plot, with a proportion of 4.1%.

### The stability of 5-aza-7-de­aza­guanine base pairs controlled by p*K* value differences of com­plementary bases   

The Watson–Crick base-pair recognition of canonical DNA and RNA constituents depends on the hydrogen-bonding donor–acceptor pattern of purine and pyrimidine nucleosides, as well as on environmental influences caused by base-to-base stacking inter­actions, helix formation and various other factors. Among these, the p*K* values of nucleobases play an important role as they control the stability and lifetime of base pairs. Recently, the role of the p*K* values of nucleobases has been discussed with regard to the origin of chemical evolution. Strong base pairs are formed when the p*K* value difference (Δp*K*) between the acceptor and donor sites of nucleobases is at least five units or more. A Δp*K* < 5 results in weaker base pairs (Krishnamurthy, 2012 ▸).

Early studies on the physical properties of 5-aza-7-de­aza-2′-de­oxy­guanosine (**2b**) showed that the nucleobase is proton­ated on the *s*-triazine moiety having a p*K_a_* value of 3.7 (Rosemeyer & Seela, 1987 ▸). Nitro­gen-1 was established as the protonation site using ^13^C NMR spectroscopy (Seela & Melenewski, 1999 ▸). The p*K_a_* value and the protonation site is different from the closely related 2′-de­oxy­guanosine (p*K_a_* value of protonation = 1.6 and deprotonation = 9.2) (Seela *et al.*, 2003 ▸), as well as to 7-de­aza-2′-de­oxy­guanosine (1.1 and 10.3) (Ramzaeva & Seela, 1996 ▸), and is a direct consequence of the transposition of the nitro­gen-7 atom to bridgehead position-5.

**Figure 8:**
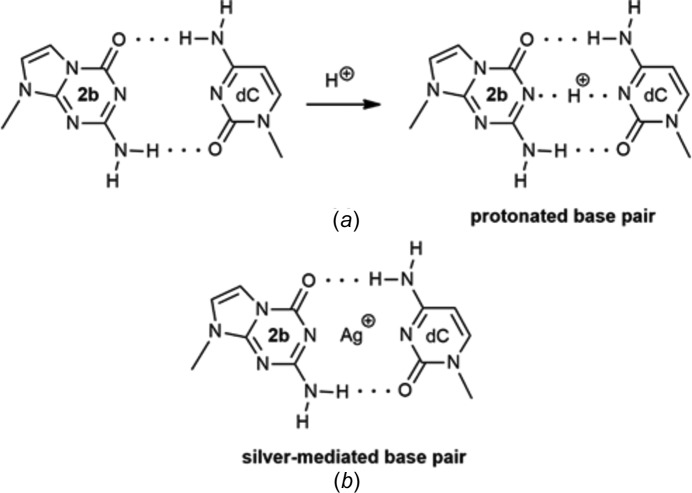
(*a*) Protonated base pair of 5-aza-7-de­aza-2′-de­oxy­guanosine (**2b**) and 2′-de­oxy­cytidine formed in acidic medium. (*b*) Silver-mediated base pair of **2b** and dC.

The positional change of the N atom leads to the absence of a proton at N1 which now becomes a proton-acceptor site and not a donor site as in 2′-de­oxy­guanosine or 7-de­aza-2′-de­oxy­guanosine. Furthermore, position-7 cannot inter­act with protons or metal ions anymore, and similar to 7-de­aza-2′-de­oxy­guanosine, Hoogsteen base pairs cannot be formed. Consequently, 5-aza-7-de­aza-2′-de­oxy­guanosine exhibits a Watson–Crick recognition face similar to that of isocytidine. Thus, the com­plementary base has to provide a proton, as observed for base pairing with guanine or isoguanine, or with protonated cytidines or cytidine derivatives which carry a proton at nitro­gen-1 (Fig. 8 ▸ *a*) (Hoshika *et al.*, 2018 ▸). Silver ions can function as proton substitutes forming metal-mediated base pairs (Fig. 8 ▸ *b*) (Guo *et al.*, 2018 ▸).

**Figure 9:**
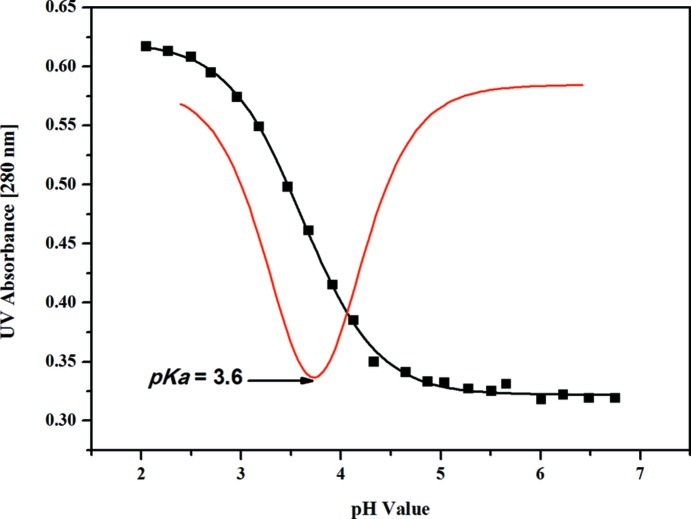
p*K_a_* value of **1** determined by UV titration (1st derivative, red line). Spectroscopic changes determined at 280 nm at different pH values (black line) were measured in 0.1 *M* sodium phosphate buffer.

As ribonucleoside **1** is a promising candidate for the synthesis of ‘all-purine’ RNA, it is of particular importance to identify the p*K_a_* value of **1** and to com­pare this value to related purine and 7-de­aza­purine nucleosides. The p*K_a_* value of com­pound **1** was determined by UV spectroscopic titration from absorbance changes at 280 nm at different pH values (Fig. S3 in the supporting information). According to Fig. 9 ▸, the p*K_a_* value of **1** is 3.6. This p*K_a_* value is almost identical to that of the corresponding 2′-de­oxy­ribonucleoside **2c** (p*K_a_* = 3.7) (Lin *et al.*, 2004 ▸) and the purine nucleoside adenosine (p*K_a_* = 3.6). Contrary to the related purine and 7-de­aza­purine nucleosides, the mol­ecule does not show a second p*K* value (for deprotonation), as the heterocyclic skeleton does not carry a proton which can be released in alkaline medium. Atom N1 is the protonation site, as was determined for 5-aza-7-de­aza­guanine 2′-de­oxy­ribonucleoside **2b** (Seela & Melenewski, 1999 ▸).

Table S3 (see supporting information) summarizes the p*K* values and protonation positions for 5-aza-7-de­aza­guanine, 7-de­aza­guanine, guanine and isoguanine nucleosides. It is obvious that the iodo substituent has no significant influence on the p*K* value of the nucleosides. However, com­pared to guanosine and 7-de­aza­guanosine, the p*K* for protonation of the unsubstituted and iodinated 5-aza-7-de­aza­guanosine base is strongly shifted and the mol­ecule is much easier to protonate. Also, the position of protonation (N1) is different from that in guanosine (N7) and 7-de­aza­guanosine (N3).

**Figure 10:**
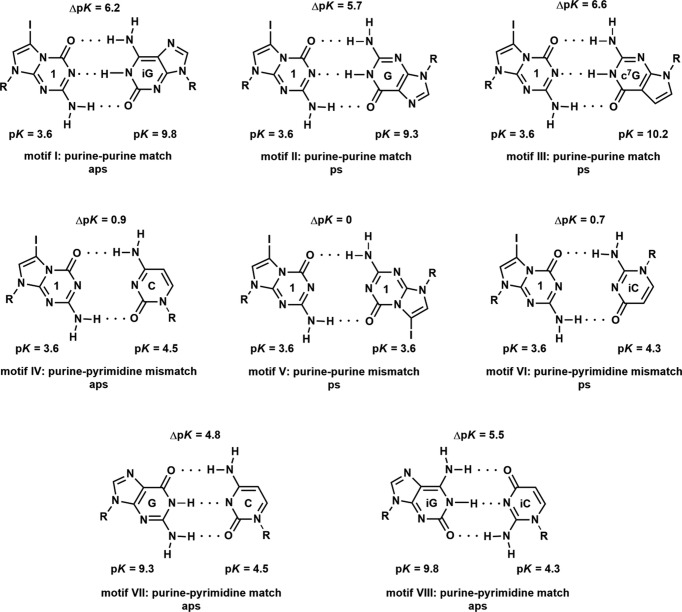
Base-pairing motifs of 5-aza-7-de­aza­guanine nucleosides, p*K_a_* values of the base-pairing nucleosides and their p*K* value difference (Δp*K*), as well as strand orientation of the respective duplex. Notes: R = β-d-ribo­furanosyl, C = cytidine, iG = isoguanosine, iC = isocytidine, G = guanosine, c^7^G = 7-de­aza­guanosine, aps = anti­parallel strands and ps = parallel strands.

It has been shown that based on p*K* value differences of nucleosides participating in base-pair formation, predictions regarding base-pair stability can be made (Krishnamurthy, 2012 ▸). To this end, the Δp*K* of base pairs of 7-iodo-5-aza-7-de­aza­guanosine with different nucleosides acting as donor or acceptor were calculated and com­pared (Fig. 10 ▸). Anti­parallel (aps) and parallel (ps) strand orientation of oligonucleotides within a duplex were considered.

The Δp*K* of the 7-iodo-5-aza-7-de­aza­guanosine–isoguanosine base pair is 6.2 (motif I, Fig. 10 ▸) referring to an aps orientation of oligonucleotides. As a result, the stability of this purine–purine base pair is expected to be higher than for the Watson–Crick purine–pyrimidine G–C base pair (Δp*K* = 4.8; motif VII, Fig. 10 ▸). A parallel arrangement of **1** with guanosine (Δp*K* = 5.7; motif II) or 7-de­aza­guanosine (Δp*K* = 6.6; motif III) also leads to stable purine–purine pairs. However, the formation of a purine–pyrimidine base pair with **1** results in a mismatch situation in an aps alignment with cytidine (Δp*K* = 0.9; motif IV) or isocytidine (Δp*K* = 0.7; motif VI). Consequently, the RNA constituent **1** is a promising candidate for the construction of stable purine–purine base pairs as building blocks for ‘all-purine’ RNA with a conventional anti­parallel orientation of the strands. Moreover, even a parallel orientation of oligonucleotides might be applicable. The 7-iodo substituent allows the introduction of reporter groups to monitor structural characteristics of aps or ps ‘all-purine’ RNA.

However, self-pairing of **1**, as demonstrated by motif V (ps alignment; Fig. 10 ▸) or even in a shifted arrangement, leads to a mismatch situation. Moreover, none of the reported crystal structures of 5-aza-7-de­aza­guanine nucleosides show self-pairing of the nucleobases (Kojić-Prodić *et al.*, 1982 ▸; Seela *et al.*, 2002 ▸; Leonard *et al.*, 2019 ▸). Only for the isolated 5-aza-7-de­aza­guanine nucleobase was self-pairing observed when one of the nucleobases forms a N3—H tautomer (Laos *et al.*, 2019 ▸). In nucleosides, as well as in nucleic acids, this arrangement is not possible as the ribose moiety is attached to N9 and the N3 atom lacks a proton.

### Functionalization of nucleoside 1 and fluorescence properties of the conjugate 3   

The ability of 7-iodo­ribonucleoside **1** to act as a target for functionalization with functional reporter groups is demonstrated by use of the Suzuki–Miyaura cross-coupling reaction (Agrofoglio *et al.*, 2003 ▸). As the benzo­furan residue is sensitive to mol­ecular crowding and the viscosity of solvents (Greco & Tor, 2007 ▸; Leonard *et al.*, 2019 ▸), this residue was chosen for functionalization of ribonucleoside **1**.

For this, com­pound **1** was used as the starting material (see Scheme 1). The synthesis of ribonucleoside **1** by nucleobase anion glycosyl­ation was reported recently, employing isobutyrylated iodo base **4** and bromo sugar **5** (Leonard *et al.*, 2019 ▸). In order to introduce the benzo­furan side chain, Suzuki–Miyaura cross-coupling of ribonucleoside **1** and benzo­furan-2-boronic acid was performed. The reaction was carried out under reflux for 30 min in a CH_3_CN/H_2_O mixture (1:1 *v*/*v*) in the presence of Pd(PPh_3_)_4_ and Na_2_CO_3_·H_2_O (see Scheme 1), yielding benzo­furan derivative **3** in 57%.

The new com­pound **3** was characterized by ^1^H and ^13^C NMR spectroscopy, as well as by ESI–TOF mass spectroscopy. ^1^H–^13^C correlated (HMBC and HSQC) NMR spectra were used to assign the ^13^C NMR signals (for spectra, see the supporting information, and for details, see the *Experimental* section).

The UV and fluorescence spectra of **3** were recorded in solvents of different polarity, and the corresponding Stokes shift and quantum yields were determined (Table S4 in the supporting information). The UV spectra of com­pound **3** show two maxima at around 312 and 327 nm in MeOH, MeCN and water (Fig. S4 in the supporting information). The maxima are bathochromally shifted (around 5 nm) in the less polar solvents dimethyl sulfoxide (DMSO) and di­methyl­formamide (DMF).

**Figure 11:**
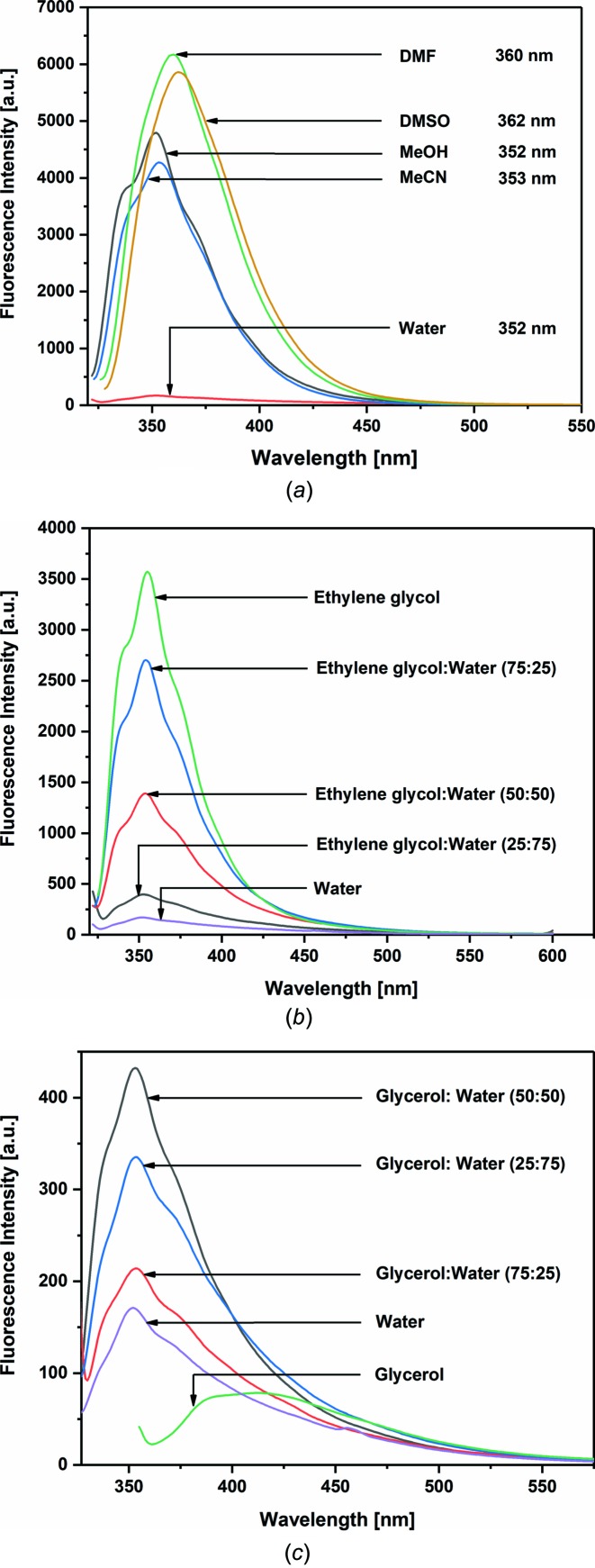
Fluorescence emission spectra of com­pound **3** (1 µ*M*) measured in (*a*) solvents of different polarity, (*b*) ethyl­ene glycol and (*c*) glycerol. Excitation wavelength of nucleoside **3** was 315 nm in ethyl­ene glycol and 345 nm in glycerol.

The fluorescence emission spectra for benzo­furan conjugate **3** in various solvents are displayed in Fig. 11 ▸(*a*). The highest quantum yields are obtained in DMF and DMSO (Table S4 in the supporting information), with emission maxima which are red-shifted com­pared to the other solvents. The fluorescence of **3** is very low in water com­pared to the other polar solvents.

In the RNA constituent **3**, the benzo­furan moiety and the 5-aza-7-de­aza­guanine base are separated by a freely rotatable ar­yl–aryl bond. The spatial positioning of both aromatic systems with respect to each other (π–π conjugation) has a direct impact on the fluorescence properties of conjugate **3**. In order to study this effect for **3**, fluorescence measurements were performed in solvents of similar polarity but different viscosity. Fig. 11 ▸(*b*) shows the fluorescence emission spectra of conjugate **3** determined in ethyl­ene glycol, water and their mixtures. The fluorescence of **3** is highest in ethyl­ene glycol and decreases with reduced viscosity of ethyl­ene glycol/water mixtures. However, the highest fluorescence is observed in a 50% glycerol–water mixture (Fig. 11 ▸ *c*). Apparently, a rotational barrier exists for the benzo­furan residue which is sensitive to solvent viscosity, a phenomenon which has been reported for other nucleoside–benzo­furan conjugates (Tan­pure & Srivatsan, 2018 ▸; Tokugawa *et al.*, 2016 ▸; Manna *et al.*, 2018 ▸).

## Conclusion and outlook   

The single-crystal X-ray structure of 7-iodo-5-aza-7-de­aza­guanosine (**1**) was determined and the geometric parameters of this RNA constituent were analyzed. This includes an *anti* conformation of the 5-aza-7-de­aza­guanine base, an *N* conformation (O4′-*endo*) of the ribose moiety and an anti­periplanar orientation of the 5′-hy­droxy group.

The crystal packing is controlled by inter­actions between the nucleobase and sugar moieties, while self-pairing of the nucleobase does not take place. The 7-iodo substituent forms a contact to oxygen-O2′ of the ribo­furanosyl moiety. A Hirshfeld surface analysis of **1** highlights the contacts of the nucleobase and sugar moieties (O—H⋯O and N—H⋯O). The concept of p*K* value differences was applied to predict base pairing of **1**. Ribonucleoside **1** has the ability to form extraordinary stable anti­parallel-stranded purine–purine pairs with isoguanosine or base pairs with guanosine displaying a parallel-strand arrangement. Related base pairs were described for ‘all-purine’ DNA (Seela *et al.*, 2001 ▸).

**Figure 12:**
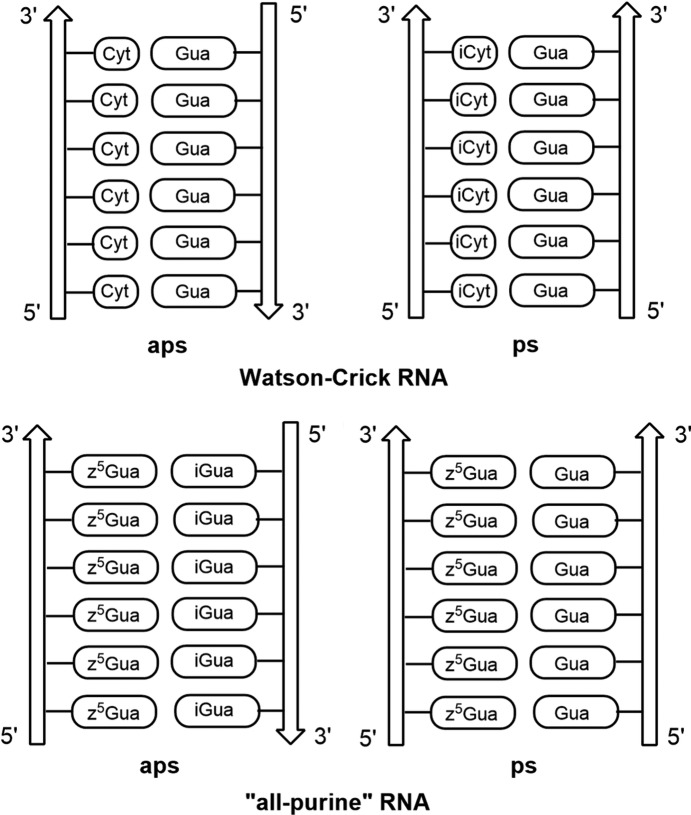
Illustration of RNA duplexes with pyrimidine–purine and purine–purine pairs with (*a*)/(*c*) anti­parallel-stranded (aps) and (*b*)/(*d*) parallel-stranded (ps) orientations. Notes: Cyt = cytosine, Gua = guanine, iCyt = isocytosine, z^5^Gua = 5-aza-7-de­aza­guanine and iGua = isoguanine.

Due to the large surface area of purine–purine pairs, base stacking in ‘all-purine’ RNA is expected to be stronger, as in canonical RNA formed by pyrimidine–purine pairs (Figs. 12 ▸ *a* and 12*b*). Furthermore, it was shown that the 7-iodo substituent of ribonucleoside **1** could be functionalized. Benzo­furan was used in this work to detect restrictions of rotational motion by fluorescence spectroscopy.

## Supplementary Material

Crystal structure: contains datablock(s) I, global. DOI: 10.1107/S2053229620004684/yf3200sup1.cif

Structure factors: contains datablock(s) I. DOI: 10.1107/S2053229620004684/yf3200Isup2.hkl

Geometric parameters of 2c, curvedness of 1, pKa values, photophysical data of 3, NMR spectra of com­pound 1. DOI: 10.1107/S2053229620004684/yf3200sup3.pdf

CCDC reference: 1950946

## Figures and Tables

**Table 1:** Experimental details

Crystal data	
Chemical formula	C_10_H_12_IN_5_O_5_
*M*_r_	409.15
Crystal system, space group	Monoclinic, *P*2_1_
Temperature (K)	100
*a*, *b*, *c* (Å)	9.1086 (2), 6.3149 (2), 11.0428 (3)
β (°)	95.585 (1)
*V* (Å^3^)	632.17 (3)
*Z*	2
Radiation type	Cu *K*α
μ (mm^−1^)	20.25
Crystal size (mm)	0.35 × 0.06 × 0.02

Data collection	
Diffractometer	Bruker KappaCCD APEXII
Absorption correction	Multi-scan (*SADABS*; Bruker, 2014 ▸)
*T*_min_, *T*_max_	0.39, 0.69
No. of measured, independent and observed [*I* > 2σ(*I*)] reflections	15087, 2216, 2144
*R*_int_	0.072
(sin θ/λ)_max_ (Å^−1^)	0.597

Refinement	
*R*[*F* ^2^ > 2σ(*F* ^2^)], *wR*(*F* ^2^), *S*	0.036, 0.097, 1.07
No. of reflections	2216
No. of parameters	205
No. of restraints	12
H-atom treatment	H atoms treated by a mixture of independent and constrained refinement
Δρ_max_, Δρ_min_ (e Å^−3^)	2.79, −1.19
Absolute structure	Flack *x* determined using 919 quotients [(*I* ^+^) − (*I* ^−^)]/[(*I* ^+^) + (*I* ^−^)] (Parsons *et al.*, 2013 ▸)
Absolute structure parameter	0.040 (7)

Com­puter programs: *SAINT* (Bruker, 2015 ▸), *SHELXT2014* (Sheldrick, 2015*a* ▸), *SHELXL2014* (Sheldrick, 2015*b* ▸), *APEX3* (Bruker, 2016 ▸) and *XP* (Bruker, 1998 ▸).

**Table 2:** Selected geometric parameters (Å, °)

I7—C7	2.081 (10)	N9—C1′	1.439 (12)
			
C4—N9—C1′	123.7 (8)	O6—C6—N1	125.1 (8)
C1′—O4′—C4′	103.7 (7)	N5—C7—I7	126.9 (7)
N2—C2—N1	117.1 (9)	O5′—C5′—C4′	110.3 (8)
			
C4′—O4′—C1′—C2′	−41.3 (9)	C1′—O4′—C4′—C3′	47.8 (9)
C4—N9—C1′—O4′	−120.6 (9)	C2′—C3′—C4′—O4′	−34.8 (9)
O4′—C1′—C2′—C3′	18.1 (9)	C3′—C4′—C5′—O5′	−172.9 (8)
C1′—C2′—C3′—C4′	9.8 (10)		

**Table 3:** Hydrogen-bond geometry (Å, °)

*D*—H⋯*A*	*D*—H	H⋯*A*	*D*⋯*A*	*D*—H⋯*A*
N2—H2*A*⋯O4′^i^	0.90 (3)	2.25 (10)	2.941 (11)	133 (11)
N2—H2*B*⋯O5′^ii^	0.90 (3)	2.04 (5)	2.905 (12)	160 (12)
O2′—H2′⋯O6^iii^	0.87 (3)	1.93 (8)	2.689 (9)	144 (12)
O5′—H5′⋯O3′^iv^	0.87 (3)	1.87 (7)	2.673 (10)	151 (13)
C1′—H1′⋯N2^v^	1.0	2.63	3.345 (15)	128
C2′—H2′1⋯O6^vi^	1.0	2.57	3.541 (12)	163

Symmetry codes: (i) 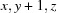; (ii) 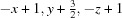; (iii) 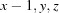; (iv) 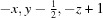; (v) 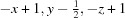; (vi) 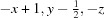.
