# Supplementary material for: 7-Iodo-5-aza-7-deazaguanine ribonucleoside: crystal structure, physical properties, base-pair stability and functionalization
Source: Acta Crystallogr C Struct Chem. 2020 Apr 29;76(Pt 5):513–23. doi: 10.1107/S2053229620004684 (PMC7199197; doi:10.1107/S2053229620004684)
Supplement: Supplementary file 3 [file c-76-00513-sup3.pdf]

Supporting Information

7-Iodo-5-aza-7-deazaguanine Ribonucleoside: Crystal Structure, Physical Properties, Base Pair Stability and Functionalization

Dasharath Kondhare[a], Simone Budow-Busse[a], Constantin Daniliuc[b] and Frank Seela[a,c*]

[a]*Laboratory of Bioorganic Chemistry and Chemical Biology, Center for Nanotechnology, Heisenbergstrasse 11, 48149 Münster, Germany*

[b]*Institut für Organische Chemie, Westfälische-Wilhelms-Universität Münster, Corrensstrasse 40, 48149 Münster, Germany*

[c]*Laboratorium für Organische und Bioorganische Chemie, Institut für Chemie, Universität Osnabrück, Barbarastrasse 7, 49069 Osnabrück, Germany*

Table of Contents

**Table S1.** Selected geometric parameters of **2c**	3
**Figure S1.** Molecular structure of compound **2c**	3
**Table S2.** ^{1}H NMR chemical shifts, coupling constants and conformation of nucleoside sugar residues	4
**Figure S2.** Curvedness of compound **1**	5
pK_a Determination by UV	5
**Figure S3.** UV-spectroscopic changes of ribonucleoside **1** at different pH values	5
**Table S3.** Summary of pK_a values of compound **1** and related nucleosides	6
**Table S4.** Photophysical data of compound **3**	7
**Figure S4.** UV-spectra of compound **3** measured in different solvents	7
**References**	8
**Figure S5-9.** NMR spectra of compound **3**	9-11

Table S1. Selected geometric parameters of 7-iodo-5-aza-7-deaza-2'-deoxyguanosine (**2c**)[a]

Geometric parameters	**2c**
Crystal system	Monoclinic
Space group	$P2_1$
Glycosylic bond length (N9-C1') [Å]	1.479(7)
Torsion angle χ (O4´-C1'-N9-C4) [°]	-139.9(6)
Relative sugar/base orientation	*Anti*
Pseudorotational phase angle *P* [°]	28.55
Maximum amplitude τ_m [°]	34.28
Sugar pucker	*N*-type, C3'-*endo*, $^3T_4$
Conformation in solution	62% *S*, 38% *N*
Torsion angle γ (O5'-C5'-C4'-C3') [°]	-172.7(4)
Relative orientation of exocyclic 5'-OH	-antiperiplanar (trans)

[a] Data were published in Leonard *et al.*, 2019.

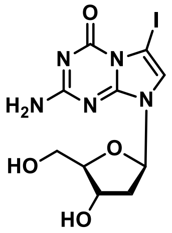

2c

Figure S1. Molecular structure of compound **2c**.

Table S2. ^{1}H NMR chemical shifts, proton-proton coupling constants and conformation of nucleoside sugar residues [a, b]

Compounds	Chemical shift/ppm							Coupling constant/Hz [*J*(HH)]					Conformation		Reference
	H-1’	H-2’	H-2’’	H-3’	H-4’	H-5’	H-5’’	1’2’	1’2’’	2’3’	2’’3’	3’4’	%N	%S	
**5-Aza-7-deazaguanine nucleosides**															
**1**	5.74 (d)	4.24 (q)	--	4.04 (q)	3.85 (q)	3.50-3.54 (ddd)	3.57-3.61 (ddd)	5.65	--	4.65	--	3.85	31	69	**1**: Leonard *et al.*, 2019
**2a**	5.78 (d)	4.28 (q)	--	4.06 (q)	3.88 (q)	3.54 (ddd)	3.57-3.64 (m)	5.60	--	4.65	--	3.85	32	68	**2a**: Leonard *et al.*, 2019
**2b**	5.34 (dd)	1.35 (ddd)	1.55 (ddd)	3.49 (dq)	2.98 (td)	2.70 (m)	--	6.1	7.6	3.2	6.0	2.9	34	66	**2b**: Rosemeyer & Seela, 1987
**2c**	6.13 (dd)	2.14 (ddd)	2.35 (ddd)	4.28 (dq)	3.78 (td)	3.52 (m)	--	6.0	7.6	3.1	6.0	2.9	34	66	**2c**: Lin *et al.*, 2004
**7-Deazaguanine nucleosides**															
**c^{7}G$_d$**	6.30 (dd)	2.32 (ddd)	2.07 (ddd)	4.28 (dq)	3.75 (td)	3.48 (m)	--	5.8	8.5	2.6	5.7	2.5	27	73	**c^{7}G$_d$**: Winkeler & Seela, 1983
**Ic^{7}G$_d$**	6.26 (dd)	2.31 (ddd)	2.05 (ddd)	4.27 (dq)	3.75 (td)	3.49 (tq)	--	5.75	8.55	2.5	5.4	2.4	25	75	**Ic^{7}G$_d$**: Ramzaeva & Seela, 1995
**c^{7}G**	5.87 (d)	4.24 (m)	--	4.03 (m)	3.80 (m)	3.54 (m)	--	5.95	--	5.45		3.65	18	82	**c^{7}G**: Seela *et al.*, 1990
**Ic^{7}G**	5.83	4.22	--	4.01	3.78	3.52	--	6.35	--	4.85		2.95	19	81	**Ic^{7}G**: Seela & Peng, 2006

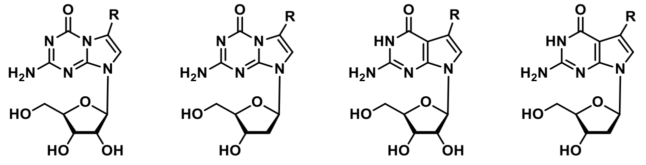

1: R = I
2a: R = H

2b: R = H
2c: R = I

c^{7}G: R = H
Ic^{7}G: R = I

c^{7}G$_d$: R = H
Ic^{7}G$_d$: R = I

[a] Measured in DMSO-*d*$_6$ at 298 K. [b] For PSEUROT calculations the coupling constants ^3J(H1’-H2’), ^3J(H2’-H3’) and ^3J(H3’-H4’) were used.

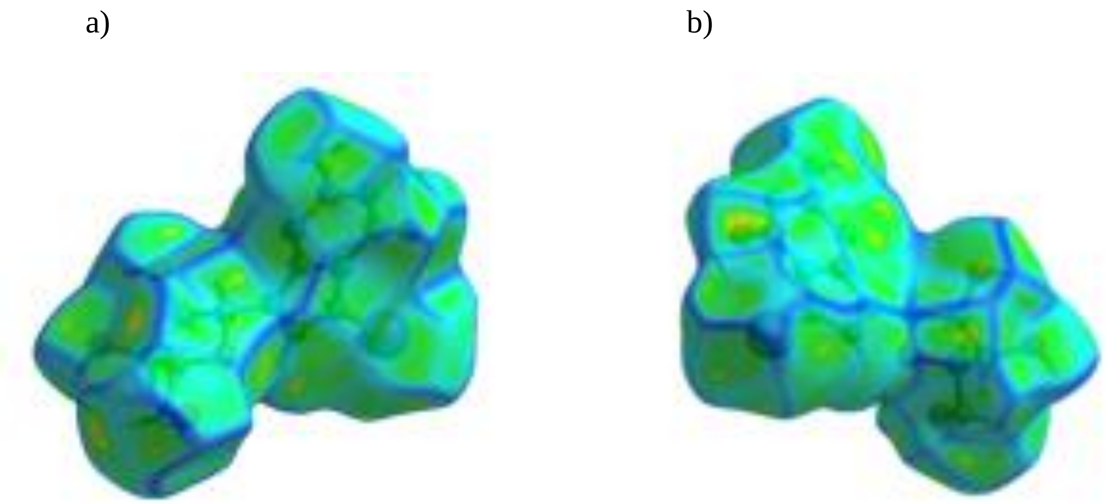

Figure S2. Curvedness of compound **1** mapped over d_{norm} (-4.0 to 0.4 Å). (a) Front and (b) back view.

pK_a Determination by UV

Ribonucleoside **1** was dissolved in 0.1 M sodium phosphate (NaH_2PO_4) buffer, pH 4.5 (250 mL). An aqueous NaOH solution (4 M) and concentrated phosphorus acid were used to adjust the pH values of the buffer. At defined pH values, the UV absorbance of nucleosides was measured (Figure S3).

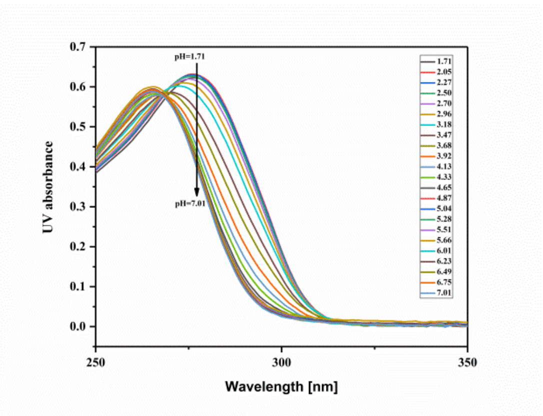

Figure S3. UV-spectroscopic changes of ribonucleoside **1** at different pH values. Spectroscopic changes were measured in 0.1 M sodium phosphate buffer.

Table S3. Summary of pK_a values of compound **1** and related nucleosides.

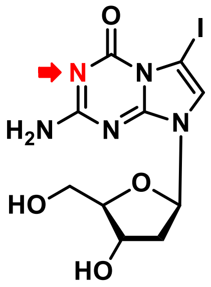<br>**3.7**<br>Lin *et al.*, 2004	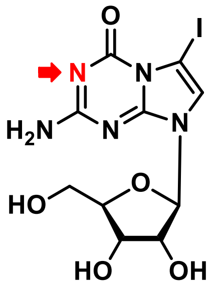<br>**3.6**	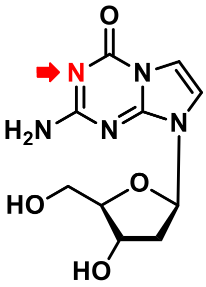<br>**3.7**<br>Rosemeyer & Seela, 1987	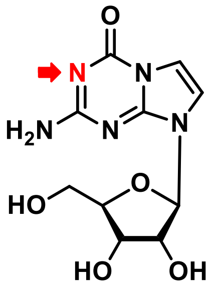<br>**3.7**
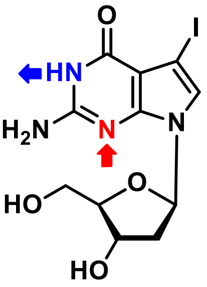<br>**< 1, 10**<br>Ramzaeva & Seela, 1996	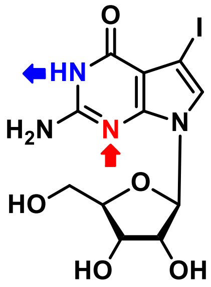<br>**< 1, 9.7**<br>Seela & Peng, 2006	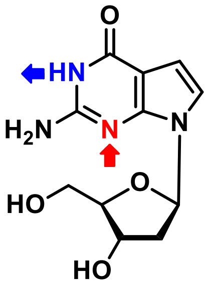<br>**1.1, 10.3**<br>Ramzaeva & Seela, 1996	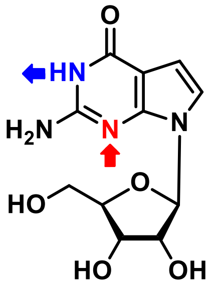<br>**1.9, 10.2**<br>Seela & Peng, 2006
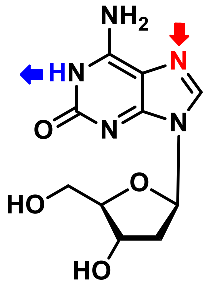<br>**3.5, 9.8**<br>Seela *et al.*, 2003	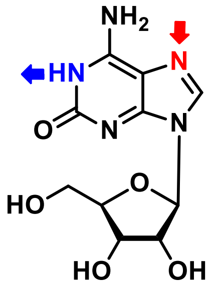<br>**3.4, 9.8**<br>Sepiol *et al.*, 1976	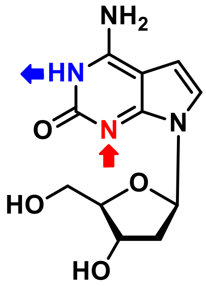<br>**4.6, 10.5**<br>Seela *et al.*, 2005	
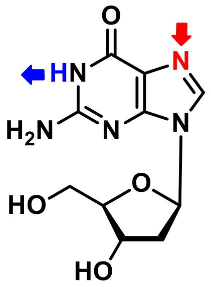<br>**1.6, 9.2**<br>Seela *et al.*, 2003	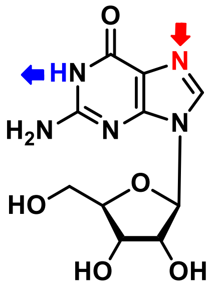<br>**1.6, 9.33**<br>Townsend *et al.*, 1972	➡ indicates protonation site<br>➡ indicates deprotonation site	

Table S4. Photophysical data of compound **3** measured in solvents of different polarity.[a]

Solvent	$\lambda_{abs, max}$ Ex [nm]	$\lambda_{max, em}$ Em [nm]	Stokes shift $(\Delta\nu)^b$ [cm^{-1}]	Φ^c
DMF	316	360	3900	0.77
DMSO	318	362	3900	0.80
MeOH	312	352	3600	0.54
MeCN	313	354	3700	0.49
Water	318	359	3600	0.10

[a] The concentration of nucleoside **3** was 1 µM. [b] The Stokes shift was calculated from the equation $\Delta E_{photon} = hc(1/\lambda_{abs,max} - 1/\lambda_{max,em})$. [c] The fluorescence quantum yields (Φ) were calculated using quinine sulfate (1 µM) in 0.1 M H_2SO_4 ($\Phi_{St} = 0.54$).

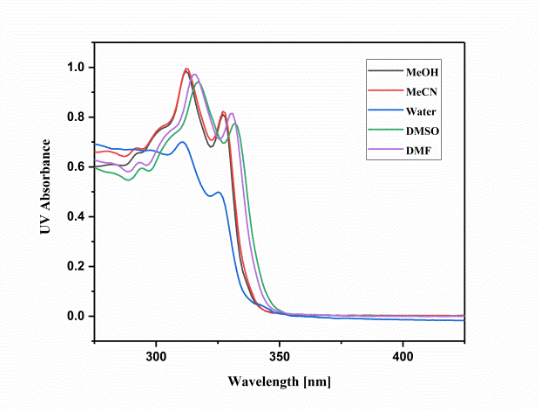

Figure S4. UV-spectra of compound **3** measured in solvents of different polarity. The nucleoside concentration was 50 µM for UV measurements.

References

Leonard, P., Kondhare, D., Jentgens, X., Daniliuc, C. & Seela, F. (2019). *J. Org. Chem.* **84**, 13313-13328.

Lin, W., Zhang, X. & Seela, F. (2004). *Helv. Chim. Acta* **87**, 2235-2244.

Ramzaeva, N. & Seela, F. (1995). *Helv. Chim. Acta* **78**, 1083-1090.

Ramzaeva, N. & Seela, F. (1996). *Helv. Chim. Acta* **79**, 1549-1558.

Rosemeyer, H. & Seela, F. (1987). *J. Org. Chem.* **52**, 5136-5143.

Seela, F., Ramzaeva, N. & Rosemeyer, H. (2003). *Science of Synthesis*, Vol. 16, edited by Y. Yamamoto, pp. 945-1108. Stuttgart: Georg Thieme Verlag.

Seela, F. & Peng, X. (2006). *J. Org. Chem.* **71**, 81-90.

Seela, F., Peng, X. & Li, H. (2005). *J. Am. Chem. Soc.* **127**, 7739-7751.

Seela, F., Soulimane, T., Mersmann, K. & Jürgens, T. (1990). *Helv. Chim. Acta* **73**, 1879-1887.

Sepiol, J., Kazimierczuk, Z. & Shugar, D. (1976). *Z. Naturforsch.* **31c**, 361-370.

Panzica, R. P., Rousseau, R. J., Robins, R. K. & Townsend, L. B. (1972). *J. Am. Chem. Soc.* **94**, 4708-4714.

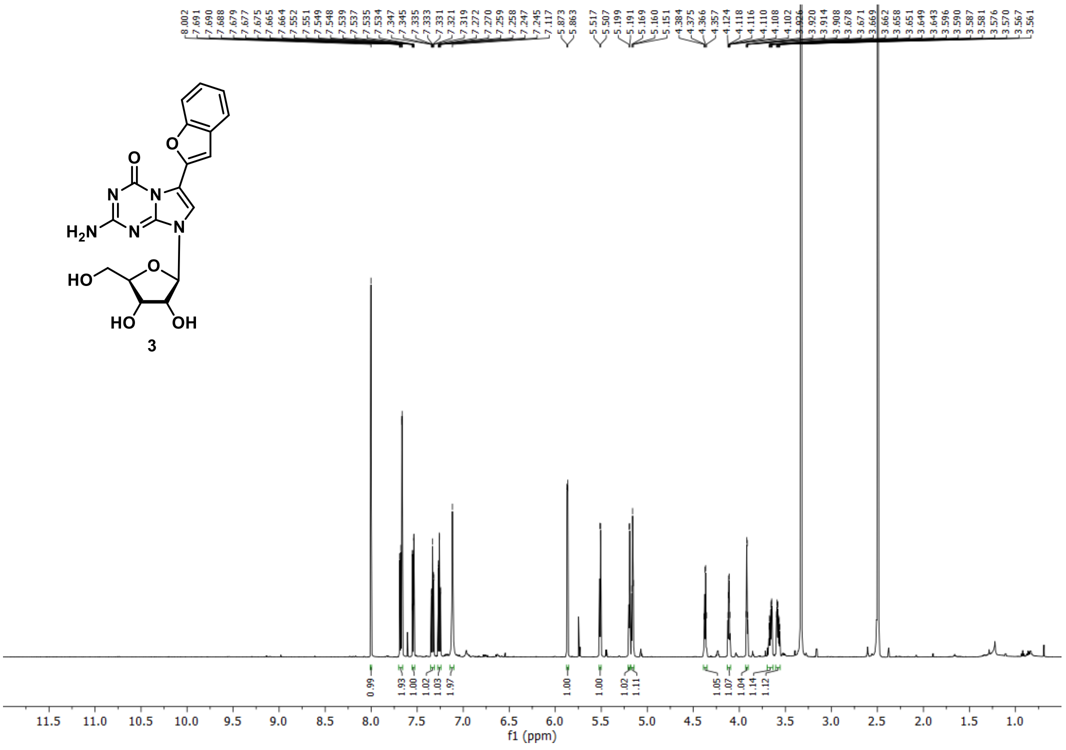

Figure S5. ^{1}H NMR spectrum of compound **3**.

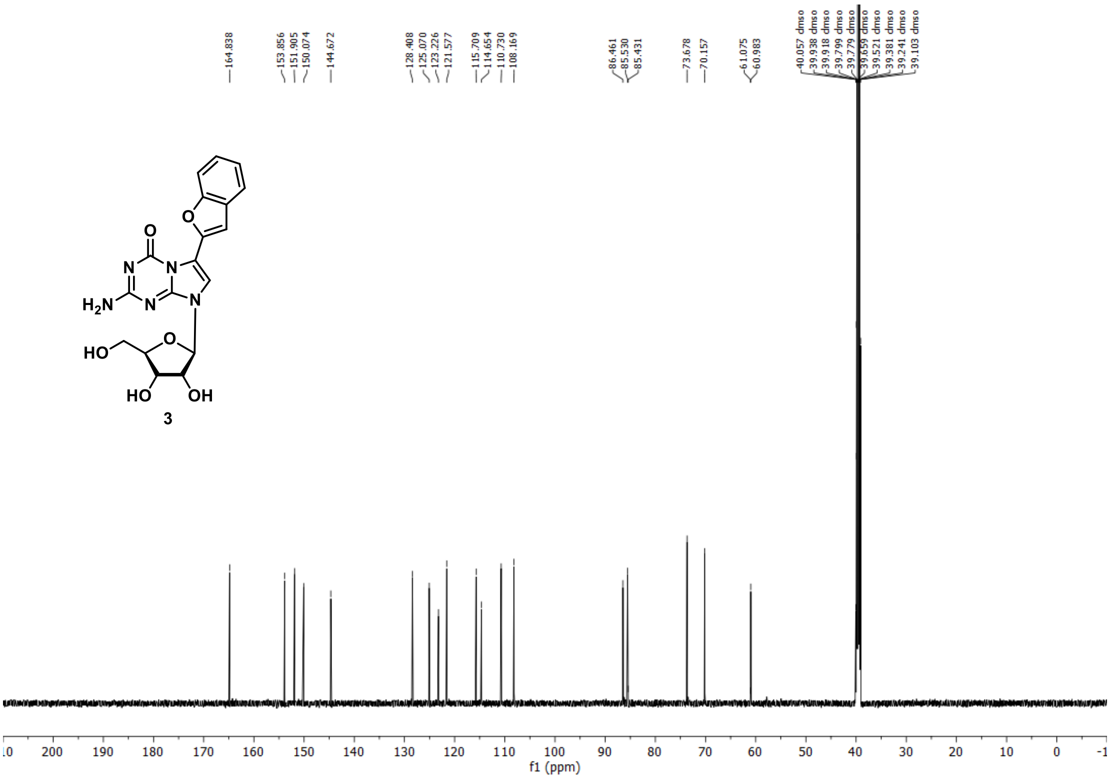

Figure S6. ^{13}C NMR spectrum of compound **3**.

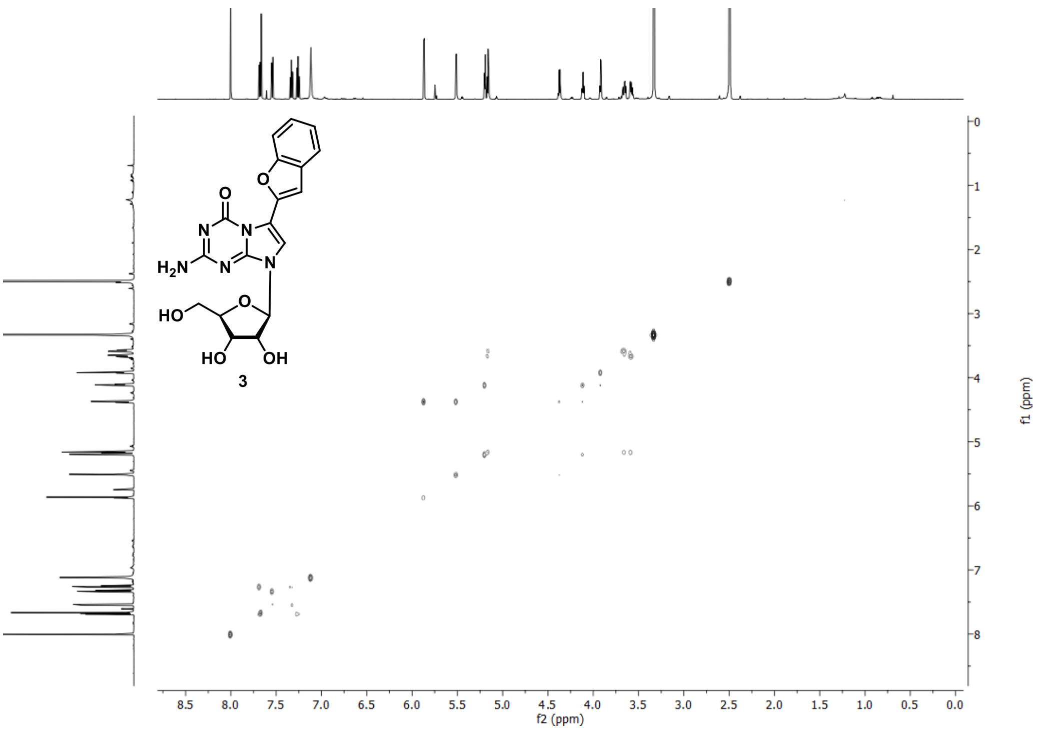

Figure S7. COSY NMR spectrum of compound **3**.

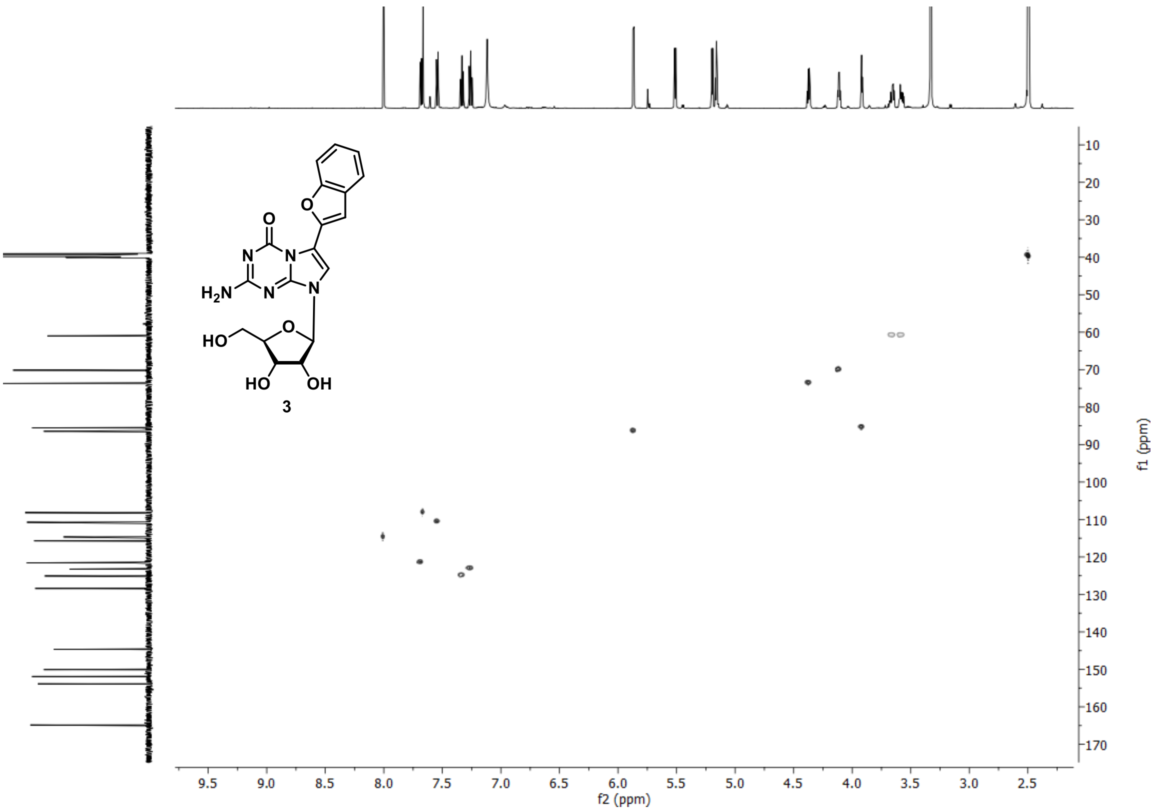

Figure S8. HSQC NMR spectrum of compound **3**.

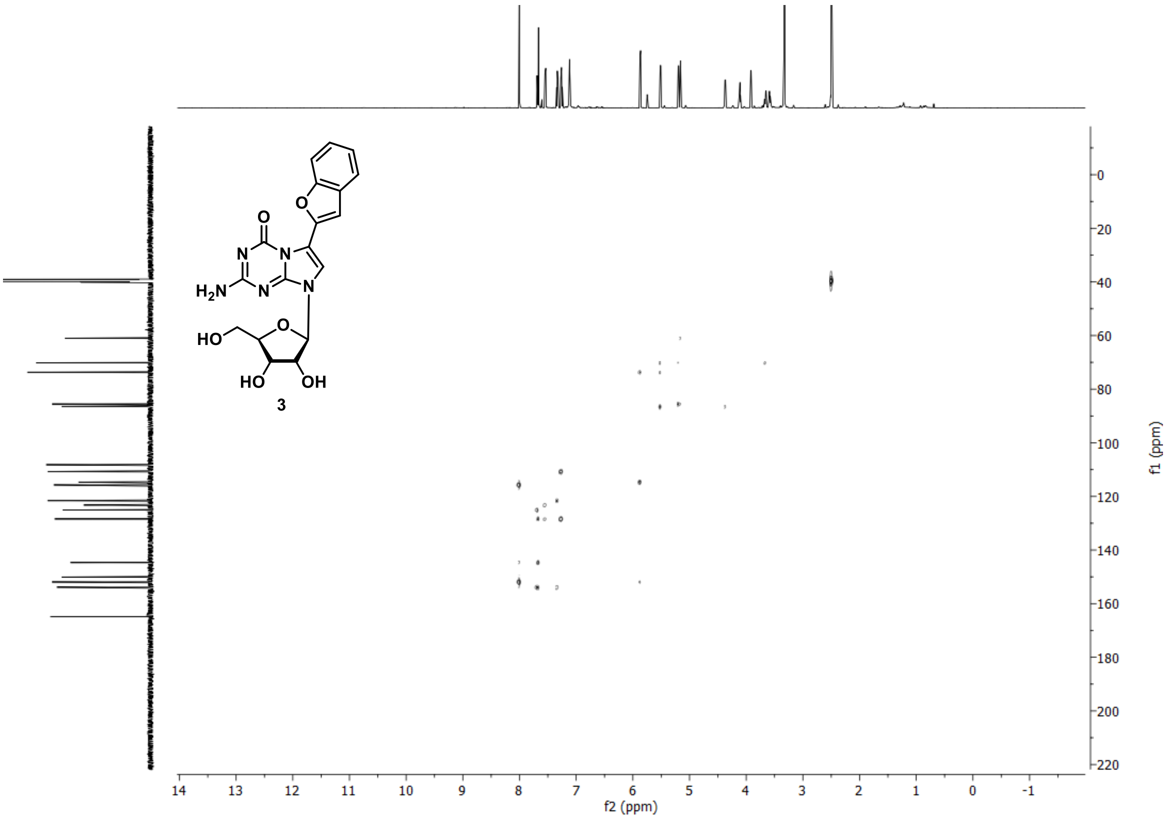

Figure S9. HMBC NMR spectrum of compound **3**.
